# Quantification of the Carboxylated LGD‐4033 Long‐Term Metabolite in Human Micro‐Dose Excretion Study Urine Samples

**DOI:** 10.1002/dta.70130

**Published:** 2026-08-03

**Authors:** Panagiotis Sakellariou, Sebastian Marcel Schröder, Jasmin Thelen, Linus Korsmeier, Sven Guddat, Christian Görgens, Andreas Thomas, Mario Thevis

**Affiliations:** ^1^ Institute of Biochemistry/Center for Preventive Doping Research German Sport University Cologne Cologne Germany; ^2^ European Monitoring Center for Emerging Doping Agents (EuMoCEDA) Cologne/Bonn Germany

**Keywords:** doping, LGD‐4033, ligandrol, metabolism, SARMs

## Abstract

Selective androgen receptor modulators (SARMs), such as LGD‐4033, are frequently detected in doping control samples and pose significant challenges for result interpretation, particularly at low concentration levels potentially arising from contamination. In this study, a quantitative method for the determination of the carboxylated long‐term metabolite (M5‐b) of LGD‐4033 in human urine was developed and applied to samples obtained from controlled micro‐dose administration studies. Urine samples from single‐ and multiple‐dose administration studies (1, 10, and 50 μg) were analyzed following enzymatic hydrolysis and solid‐phase extraction using LC‐HRMS/MS. Quantification was enabled by the use of a recently synthesized certified reference material, allowing, for the first time, the generation of quantitative excretion data for long‐term metabolite under micro‐dose conditions. The results demonstrated the rapid formation of long‐term metabolite and a clear dose‐dependent increase in urinary concentrations. Extended detection windows were observed for all dosing regimens, with long‐term metabolite remaining detectable for several days after single‐dose administration and for substantially longer periods following consecutive administration (5 days). Accumulation effects during multiple‐dose intake resulted in sustained urinary concentrations and prolonged detectability. The quantitative data generated in this study provide an important basis for improved interpretation of adverse analytical findings involving LGD‐4033 and support a more evidence‐based distinction between different intake scenarios.

## Graphical abstract

A validated LC‐HRMS/MS method enabled the first quantitative determination of the LGD‐4033 long‐term metabolite (M5‐b) in urine following controlled micro‐dose administration. The generated urinary reference data support the interpretation of adverse analytical findings and anti‐doping result management.

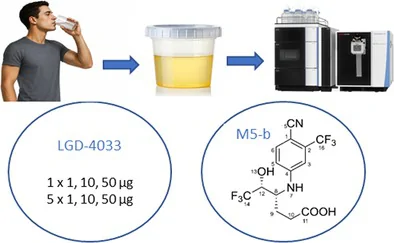

## 1 Introduction

Selective androgen receptor modulators (SARMs) represent a class of non‐steroidal compounds designed to selectively activate the androgen receptor, thereby promoting anabolic effects while minimizing undesirable androgenic side effects [1]. Among these, LGD‐4033 (Ligandrol) is an orally active SARM that has been investigated for the treatment of muscle wasting conditions at doses of approximately 1 mg per day [2, 3]. However, higher doses (e.g., 10 mg per day) are not recommended and have been associated with adverse effects, including hepatotoxicity [4]. Despite the lack of clinical approval for therapeutic use, LGD‐4033 has been detected in black‐market products as well as in athlete samples [5, 6, 7, 8, 9]. Owing to its performance enhancing properties and misuse in sport, LGD‐4033 is included in the World Anti‐Doping Agency (WADA) Prohibited List, and its detection in doping control samples has increased in recent years, with numerous adverse analytical findings (AAFs) being reported [10, 11].

An important challenge in anti‐doping practice is the unintentional ingestion of (or exposure to) prohibited substances, for example, through contaminated dietary supplements. Even trace‐level contamination may result in AAFs, creating significant issues for both athletes and result management authorities. While advances in analytical methodologies have substantially improved detection sensitivity and extended retrospective testing capabilities, they have also increased the likelihood of identifying minute quantities originating from inadvertent exposure. Reports from the United States Anti‐Doping Agency (USADA) have demonstrated that certain dietary supplements were either contaminated with or deliberately adulterated with LGD‐4033, potentially leading to AAFs in recent years [12]. Under the World Anti‐Doping Code and its principle of strict liability, athletes are required to provide compelling evidence that such findings are the result of unintentional exposure rather than deliberate use [13]. Although quantitative data on LGD‐4033 contamination are scarce, contamination levels in the ng/g range (e.g., a contaminated supplement with approximately 50 ng/g LGD‐4033) could plausibly result in cumulative daily exposures in the μg range, for example, following repeated consumption of a contaminated supplement.

Extensive research has been conducted to elucidate the metabolism of LGD‐4033 in humans. In vivo studies have identified a broad range of phase I metabolites, including mono‐, di‐, and tri‐hydroxylated derivatives as well as epimerization products. Among these, the carboxylated metabolites (M5 isomers) have consistently been recognized as the most relevant long‐term metabolites, exhibiting detection windows extending over several days to weeks and therefore representing key analytical targets in doping control [6, 8, 14, 15, 16]. To enhance the interpretation of analytical findings, controlled human excretion studies employing micro‐dose administrations (1, 10, and 50 μg) were conducted. These investigations revealed a complex metabolic profile and demonstrated time‐dependent changes in metabolite patterns. In particular, shifts in the ratios between LGD‐4033, its epimer M1, and the long‐term metabolites M5‐a and M5‐b were observed [17]. The evaluation of these dynamic relationships provided valuable insights into the timing and extent of substance intake. Accordingly, the combined interpretation of analyte concentrations and metabolite ratios represents a promising tool for distinguishing between recent low‐level exposure and the prolonged elimination of higher doses, thereby supporting the assessment of contamination related scenarios. More recent studies further support that the combined evaluation of early‐stage excreted and long‐term metabolites can improve the differentiation between intentional administration, micro‐dosing, and inadvertent exposure [18].

Building upon these developments, the present study aims to reanalyze urine samples obtained from previously conducted LGD‐4033 micro‐dose administration studies by enzymatic hydrolysis and solid phase extraction (SPE) in order to quantify the carboxylated long‐term metabolite of LGD‐4033 ((4R,5R)‐4‐{[4‐cyano‐3‐(trifluoromethyl)‐phenyl]amino}‐6,6,6‐trifluoro‐5‐hydroxyhexanoic acid) (M5‐b, Figure 1), using the recently synthesized certified reference material [17, 19]. For this purpose, a validated quantitative method based on liquid chromatography‐high resolution mass spectrometry (LC‐HRMS/MS) was applied. The determination of urinary M5‐b concentrations under controlled micro‐dose conditions is expected to generate valuable reference data, thereby improving the interpretation of AAFs and supporting a more reliable distinction between deliberate doping and inadvertent exposure scenarios.

**FIGURE 1:**
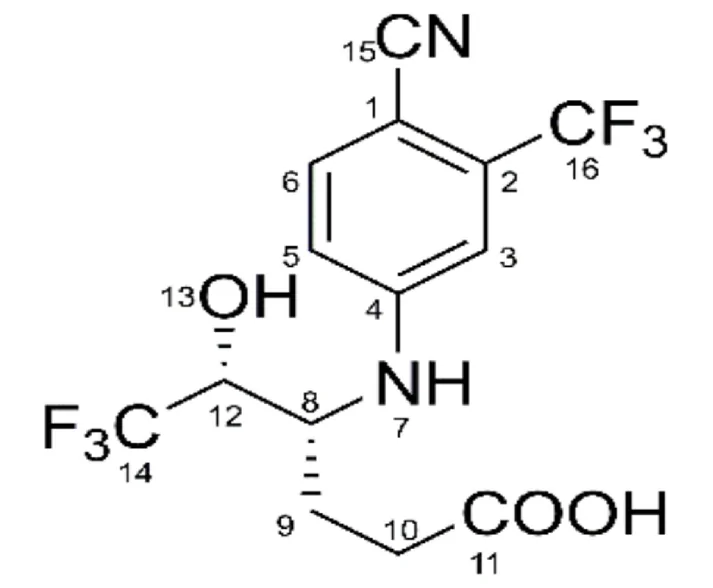
Chemical structure of the carboxylated long‐term metabolite of LGD‐4033 (M5‐b) [19].

## 2 Material and Methods

### 2.1 Reference Material, Chemicals, and Consumables

The certified reference material of the LGD‐4033 carboxylated long‐term metabolite M5‐b was synthesized and characterized as described previously and delivered by WADA [19]. The arylpropionamide‐derived SARM S‐24 was used as an internal standard (ISTD) and was synthesized in‐house as described previously [20]. Acetonitrile (ACN), isopropanol and disodium hydrogen phosphate monohydrate (Na_2_HPO_4_·H_2_O) were obtained from VWR Chemicals (Radnor, PA, USA). Ammonium acetate, ammonium hydroxide, hydrochloric acid, formic acid and sodium dihydrogen phosphate (NaH_2_PO_4_) were purchased from Merck (Darmstadt, Germany). Methanol (MeOH) was obtained from J.T.Baker (Phillipsburg, NJ, USA). β‐Glucuronidase ( *Escherichia coli* ) was purchased from Kura Biotech (Rancho Dominguez, CA, USA). Ultrapure water was produced using an Arium Pro system from Sartorius (Göttingen, Germany). SPE was performed using Chromabond HR‐XCW 96‐well plates (45 μm/25 mg) or Chromabond HR‐XCW cartridges (1 mL/45 μm/25 mg) obtained from Macherey‐Nagel (Düren, Germany). Deep‐well collecting plates (2 mL) were also obtained from the same supplier. 96‐well plates for LC–MS analysis were obtained from Thermo Fisher Scientific (Bremen, Germany).

### 2.2 Excretion Studies

Urine samples analyzed in the present study originated from previously conducted controlled human administration studies involving micro‐doses of LGD‐4033, as described elsewhere [17]. Briefly, healthy male volunteers from the general population received either single or multiple oral administrations (spiked drinking yoghurt) of LGD‐4033 at doses of 1, 10, or 50 μg. For single‐dose studies, urine samples were collected prior to administration and continuously during the first 48 h, followed by daily sampling until target analytes were no longer detectable. For multiple‐dose studies, participants received repeated daily administrations over five consecutive days. Urine samples were collected prior to administration and continuously during the first 48 h, followed by four samples per day during the subsequent 72 h, and thereafter one sample per day until target analytes were no longer detectable. All samples were stored at −20°C until analysis. Written informed consent to participate and to the publication of their data in anonymized form was obtained from all participants, and the study was approved by the Ethics Committee of the German Sport University Cologne (No. 040/2020) prior to its initiation.

### 2.3 Sample Preparation

Urine samples were processed using a SPE‐based workflow, using a Hamilton Vantage fully automated liquid handling system (Hamilton Company, Reno, NV, USA), according to the procedure described below. Samples anticipated to contain concentrations above the validated calibration range were diluted appropriately prior to sample preparation. Using the automated system, 1 mL of each urine sample was transferred into a deep‐well plate and spiked with 10 μL of ISTD (S‐24, 10 ng/mL). Enzymatic hydrolysis was performed by adding 15 μL of β‐glucuronidase and 400 μL of phosphate buffer (0.4 M, pH 7.0), followed by mixing and incubation at 50°C for 20 min. After hydrolysis, samples were acidified with 60 μL of hydrochloric acid (6 N) and mixed with 200 μL of isopropanol. Sample clean‐up was performed using Chromabond HR‐XCW 96‐well SPE plates. Prior to sample loading, the sorbent was conditioned with 1 mL of isopropanol and equilibrated with 1 mL of ultrapure water. Following application of the acidified samples, the cartridges were washed sequentially with 1 mL of ultrapure water and 0.5 mL of a water/methanol mixture (60:40, v/v). Analytes of interest were eluted into a 96‐well plate with 0.5 mL of a solution consisting of 5% ammonium hydroxide in methanol (5:95, v/v). The eluates were evaporated to dryness under a stream of air. The residues were reconstituted in 100 μL of acetonitrile/water/formic acid (20:79:1, v/v/v) prior to LC‐HRMS/MS analysis.

### 2.4 LC‐HRMS/MS

The LC‐HRMS/MS analysis was performed using a Vanquish Flex HPLC system coupled to an Orbitrap Exploris 480 mass spectrometer (Thermo Fisher Scientific, Bremen, Germany). Chromatographic separation was achieved using an EC 4/2 Nucleodur C‐18 Pyramid precolumn (3 μm, 4 × 2 mm) and an EC 50/2 Nucleodur C‐18 Pyramid analytical column (1.8 μm, 50 × 2 mm) obtained from Macherey‐Nagel (Düren, Germany). The mobile phase consisted of 5 mM ammonium acetate buffer containing 0.1% acetic acid (pH 3.5) as solvent A and ACN as solvent B. A linear gradient elution was applied with a total run time of 16.5 min. The initial conditions were set to 25% B at a flow rate of 200 μL/min, increased to 40% B within 5 min, and further to 100% B over the following 6 min. The organic phase was maintained at 100% B for 1 min at 200 μL/min and an additional 1 min at 350 μL/min, followed by re‐equilibration at 25% B for 3 min at 350 μL/min and 0.5 min at 200 μL/min. The injection volume was 5 μL. Mass spectrometric detection was performed in negative electrospray ionization mode with an ionization voltage of −3000 V and a transfer tube temperature of 350°C. Full‐scan experiments were acquired over an m/z range of 100–800 at a resolution of 60,000 full width at half maximum (FWHM). Targeted MS/MS experiments were recorded with a resolution of 30,000 FWHM, via higher‐energy collisional dissociation (HCD) fragmentation, for M5‐b and ISTD as shown in Table 1. The normalized collision energy was set to 30%. Precursor ions were isolated using a 1.5 *m/z* window. Nitrogen was used as collision gas and supplied by a CMC nitrogen generator (Eschborn, Germany). The mass spectrometer was routinely calibrated according to the manufacturer's recommendations.

**TABLE 1:** LC‐HRMS/MS identification parameters for M5‐b and ISTD. Product ions used for the quantification are marked in bold.

Analyte	Formula of deprotonated molecule	Exact mass/charge ratio (*m/z*)	Retention time (min)	Exact mass/charge ratio of product ions (*m/z*)
LGD‐4033 M5‐b	C_14_H_11_F_6_N_2_O_3_^−^	369.0679	7.1	**281.0543**
				237.0645
				253.0594
S‐24 (ISTD)	C_18_H_13_O_3_N_2_F_6_^−^	381.0868	9.4	**241.0594**

### 2.5 Data Evaluation

Data evaluation was performed using Thermo Xcalibur software (Version 4.0.27.10, 2015). Peak areas of the target analytes were normalized using the corresponding peak area of the ISTD (S‐24) to correct for analytical variability. A *t*‐test was performed to assess the significance of the intercept after linear regression. The intercept was not statistically different from zero (*t* = −0.67, *p* = 0.53); thus, quantitative estimation via single point calibrator is enabled. Two quality control (QC) samples at 0.05 and 1 ng/mL were analyzed within each analytical batch. For quantification, measured concentrations below 0.5 ng/mL were evaluated using the low‐level QC, while concentrations above this value were assessed using the high‐level QC, ensuring reliable quantification across the investigated concentration range. In cases where measured concentrations exceeded the validated calibration range, samples were diluted accordingly to fall within the validated range, and final concentrations were calculated by applying the respective dilution factor. Where appropriate, analyte concentrations were additionally adjusted based on the specific gravity (SG) of the urine samples, in accordance with the WADA TD2022DL [21]. For data visualization, excretion profiles were generated using predefined time intervals to account for nonuniform sampling times across volunteers. Samples with concentrations below the limit of identification (LOI) were not included in the evaluation. Mean concentrations were calculated per time interval, and the number of data points, as well as the mean, maximum, and minimum values per time interval, are provided in the Supporting Information (Tables S1–S6).

### 2.6 Method Validation

The method was validated for the detection and quantification of the LGD‐4033 long‐term metabolite M5‐b in human urine, according to current WADA criteria for confirmation procedures. All validation samples were prepared following the preparation procedure as described above. Blank urine samples obtained from male and female volunteers with varying SG and pH values were used throughout the validation.

Selectivity was assessed by analyzing 10 blank urine samples (five male and five female) spiked only with the ISTD, while the assay's reliability was evaluated in these 10 different urine samples fortified with M5‐b at a concentration of 1 ng/mL. Similarly, six of these urine samples (three male and three female) were fortified with M5‐b at five different concentrations (0.50, 0.25, 0.10, 0.05, and 0.01 ng/mL) and analyzed to evaluate the LOI, considering retention time, accurate mass, and relative ion abundances in accordance with WADA TD2023IDCR criteria [22]. The dynamic range was determined by preparing three calibration curves in three different urine samples spiked at seven concentration levels (0.01, 0.05, 0.10, 0.25, 0.50, 1, and 2 ng/mL) and at four replicates per level. The limit of detection (LOD) was determined as the value of the sigmoidal fit function at a 95% detection rate. Moreover, potential carry‐over was assessed during LC‐HRMS/MS analysis, by analyzing a blank sample extract following the injection of a urine sample fortified with M5‐b at a concentration of 2 ng/mL. Repeatability (intraday precision, *n* = 5) and intermediate precision (interday precision, *n* = 10) were evaluated in one spiked urine sample at two concentration levels (0.1 and 1 ng/mL). Intraday precision was assessed from five replicate analyses performed on the same day, whereas intermediate precision was determined from replicate analyses performed over two separate days (five replicates per day). Robustness was demonstrated by analyzing urine samples processed by two different analysts and by manual sample preparation using SPE cartridges, instead of an SPE 96‐well plate, under otherwise identical conditions.

## 3 Results and Discussion

### 3.1 Assay Characterization

The method fulfilled the current WADA criteria and proved suitable for the detection and quantification of the LGD‐4033 long‐term metabolite M5‐b in human urine (Table 2). No interfering signals were observed in any of the analyzed blank urine samples at the retention time of M5‐b, confirming the selectivity of the method. The identification capability was successfully demonstrated in all fortified samples at 1 ng/mL. The LOI experiments demonstrated that M5‐b could be reliably identified at concentrations down to 0.01 ng/mL, fulfilling the WADA criteria for retention time and relative ion abundances. The method exhibited a consistent dynamic range across all tested calibration curves, covering the concentration range of 0.01–2.00 ng/mL, with acceptable linearity (*R* ^2^ > 0.998). The observed linearity enabled the quantitative analysis of excretion study samples using single‐point calibration. Two QC levels at 0.05 and 1.00 ng/mL were included in each analytical batch for quantification. No relevant carry‐over was observed, as blank samples analyzed after high‐concentration injections (2 ng/mL) did not show detectable signals of M5‐b. Intermediate precision was demonstrated at both investigated concentration levels of 0.10 and 1.00 ng/mL, showing acceptable intraday (≤ 15.7%) and interday (≤ 16.6%) variability. The method proved robust when samples were processed by different analysts and when manual SPE cartridge‐based extraction was performed.

**TABLE 2:** Method validation results for LGD‐4033 M5‐b.

Selectivity	100%		*n* = 10
Reliability	100%	At 1 ng/mL	*n* = 10
LOI	0.01 ng/mL		*n* = 6
Carry‐over	0%	At 2 ng/mL	*n* = 1
Linearity (dynamic range)	*R* ^2^ = 0.9986	0.01–2 ng/mL	*n* = 4
LOD	0.01 ng/mL		*n* = 6
Intraday precision	8.3%	At 0.1 ng/mL	*n* = 5
Intraday precision	15.7%	At 1 ng/mL	*n* = 5
Interday precision	9.9%	At 0.1 ng/mL	*n* = 10
Interday precision	16.6%	At 1 ng/mL	*n* = 10

### 3.2 Single‐Dose Studies

Following single oral administration of LGD‐4033 at doses of 1, 10, and 50 μg, the long‐term metabolite M5‐b was consistently detected in urine samples of all volunteers. Rapid formation of M5‐b was observed, with maximum urinary concentrations (*C*_max_) occurring within approximately the first 2–9 h after administration across all dose levels. A clear dose‐dependent increase in M5‐b concentrations was evident, with peak mean concentration estimated at 0.33 ng/mL at 1 μg, 2.33 ng/mL at 10 μg and up to 29.1 ng/mL at 50 μg (Figure 2). The *C*_max_ for each individual ranged from 0.2 to 0.6 ng/mL at 1 μg, from 2.1 to 3.8 ng/mL at 10 μg, and from 3.9 to 82.6 ng/mL at 50 μg. Following the initial peak, M5‐b concentrations declined rapidly within the first 24 h, after which a slower elimination phase was observed up to 48 h after administration. Despite the low administered doses, M5‐b remained detectable over extended periods, with detection windows reaching up to 96 h for the lowest dose, 192 h for 10 μg, and 312 h for the highest dose. These findings confirm the high diagnostic value of M5‐b as a long‐term metabolite, even after micro‐dose administration. Although *C*_max_ and detection window of M5‐b varied between the five individuals, a comparable excretion pattern was observed. Overall, the quantitative data demonstrate a clear dose‐dependent relationship between administered dose and urinary concentrations, while also highlighting the importance of incorporating temporal information into the interpretation of analytical findings. These results are in agreement with previous studies reporting prolonged detection windows for carboxylated metabolites of LGD‐4033 and further support their relevance for doping control purposes. Notably, the availability of quantitative data enables a more refined and evidence‐based interpretation compared to earlier qualitative approaches.

**FIGURE 2:**
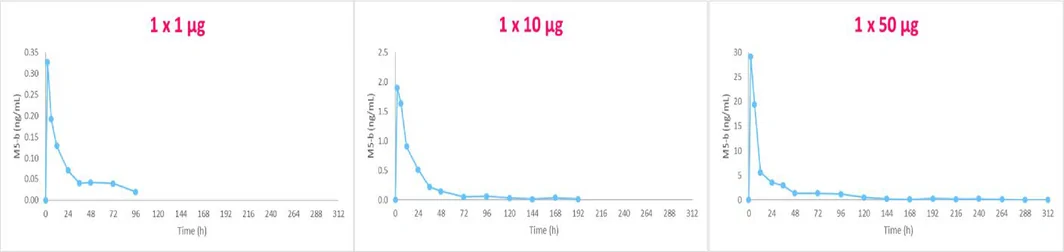
Urinary excretion profiles of mean M5‐b concentrations following single oral micro‐dose administration of LGD‐4033 (1, 10, and 50 μg).

### 3.3 Multi‐dose Studies

Multiple oral administration of LGD‐4033 over five consecutive days (1, 10, and 50 μg per day) resulted in a clear accumulation of the long‐term metabolite M5‐b in urine samples, especially after the third administration day (48 h), as presented in Figure 3. In contrast to the single‐dose study, elevated urinary concentrations were observed throughout the dosing period, with multiple local maxima observed from 4 to 120, 4 to 96, and 4 to 120 h after the first administration of 1, 10, and 50 μg, reflecting consecutive administrations and progressive accumulation. The *C*_max_ were observed shortly after the dosing phase and exhibited a clear dose‐dependent pattern, especially for the first two administration days, with peak mean concentration estimated at 0.98 ng/mL at 1 μg, 4.75 ng/mL at 10 μg, and 31.9 ng/mL at 50 μg (Figure 3). The *C*_max_ for each individual ranged from 1.0 to 3.9 ng/mL at 1 μg, from 4.1 to 17.2 ng/mL at 10 μg, and from 15.3 to 240.7 ng/mL at 50 μg. Compared to single‐dose administration, substantially prolonged detection windows were observed, with M5‐b remaining detectable for up to 264 h at 1 μg, 384 h at 10 μg, and 840 h at 50 μg after the first administration. After cessation of administration, M5‐b concentrations declined gradually, exhibiting a slower elimination phase compared to the single‐dose study. Notably, elevated concentration levels were maintained for several days after administration, indicating that repeated micro‐dose intake leads to sustained metabolite presence in urine. Despite the overall dose‐dependent trends, variability between individual time points was observed, particularly during the dosing phase, likely reflecting differences in urine sampling times and accumulation kinetics. Overall, the results demonstrate that repeated administration results in higher baseline concentrations, sustained urinary levels, and extended detection windows of M5‐b compared to single‐dose intake. These findings highlight the increased sensitivity of detecting repeated intake scenarios and underline the importance of combining concentration data with temporal information for reliable interpretation in anti‐doping contexts.

**FIGURE 3:**
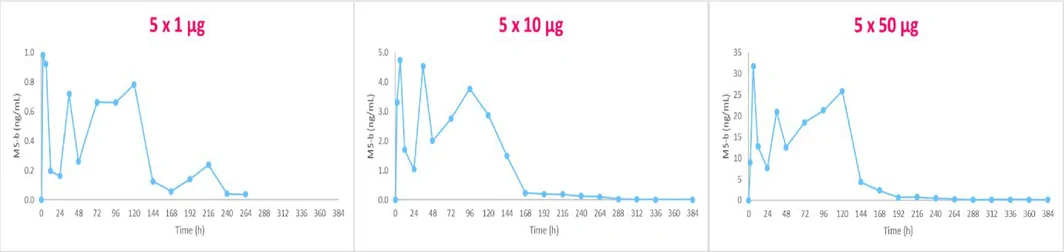
Urinary excretion profiles (up to 384 h) of mean M5‐b concentrations following multiple oral micro‐dose administration of LGD‐4033 (5 × 1, 5 × 10, and 5 × 50 μg).

### 3.4 Interpretation of M5‐b Excretion Profiles

The present study provides, for the first time, quantitative data on the urinary excretion of the LGD‐4033 long‐term metabolite M5‐b, following controlled micro‐dose administration. The availability of a certified reference material enabled the transition from previously reported qualitative or semiquantitative approaches to a fully quantitative analytical strategy, thereby significantly enhancing the interpretative value of analytical findings in doping control [19]. The results confirm that M5‐b represents a highly sensitive and long‐lasting marker of LGD‐4033 intake, even at micro‐dose levels, with extended detection windows observed in both single‐ and multi‐dose administration studies [14, 17]. A direct comparison of the excretion profiles obtained after single‐ and multi‐dose administration reveals distinct differences in both concentration levels and elimination behavior of M5‐b. While single‐dose administration resulted in a rapid increase followed by a decline in urinary concentrations, consecutive administration led to an evidence of metabolite accumulation and sustained elevated levels throughout the dosing period. This accumulation effect was evident across different investigated dose levels and resulted in higher concentrations and substantially prolonged detection windows. For better visualization of the interindividual variability, the individual urinary excretion profiles of all volunteers without grouping into predefined time intervals are provided in the Supporting Information (Figures S1 and S2).

From a result management perspective, the quantitative data generated in this study provide additional information beyond mere detection. While previous studies have demonstrated that metabolite ratios can assist in estimating the timing of intake, the incorporation of absolute concentration data allows for a more comprehensive evaluation of potential exposure scenarios [17]. However, partial overlap between late elimination phases is an important limitation, indicating that concentration data alone are insufficient for unequivocal discrimination between different intake scenarios. Nevertheless, the data suggest that higher urinary concentrations of M5‐b, exceeding approximately 250–500 ng/mL, are less likely to be associated with inadvertent exposure scenarios comparable to the low‐dose exposure scenarios investigated in the present study. In particular, concentrations substantially exceeding the range observed after controlled micro‐dose administration would be difficult to reconcile with contamination alone and may instead indicate repeated or higher‐dose intake. Therefore, a combined evaluation of concentration levels, temporal excretion patterns, additional analytical markers, and reference data for alternative routes of administration, such as transdermal exposure, is recommended to improve the significance of result interpretation [18, 23]. Overall, the findings of this study contribute to a more evidence‐based and nuanced interpretation of LGD‐4033 AAFs. The generated quantitative reference data for M5‐b can support anti‐doping authorities in assessing the plausibility of contamination scenarios and in distinguishing them from intentional doping practices. Future studies should aim to further refine interpretative models by integrating additional qualitative data for other metabolites, thereby expanding the available dataset.

## 4 Conclusions

This study presents the first quantitative reference data on the long‐term metabolite M5‐b of LGD‐4033 following controlled micro‐dose administration. The application of a validated LC‐HRMS/MS method, supported by a certified reference material, enabled reliable quantification across a wide concentration range and represents a significant advancement over previously reported qualitative approaches. The results confirm that M5‐b is a highly sensitive and long‐lasting marker of LGD‐4033 intake, with extended detection windows observed even at very low doses. Clear differences between single‐ and multiple‐dose administration were identified, with consecutive intake leading to accumulation effects, sustained urinary concentrations, and prolonged detectability. The findings emphasize that a comprehensive evaluation combining concentration levels, temporal excretion profiles, and additional analytical markers is required for robust differentiation between intentional administration, repeated intake, and inadvertent exposure. Importantly, the generated quantitative data provide valuable support for result management processes and may assist antidoping authorities in assessing the plausibility of contamination scenarios. While extremely high urinary concentrations are unlikely to be explained by accidental exposure, further studies are warranted to refine interpretative frameworks and expand the available dataset across different administration patterns.

## Author Contributions

M.T. conceptualized the project. A.T., P.S., S.G., C.G., S.M.S., and L.K. developed and optimized the LC‐HRMS/MS detection method. P.S., S.M.S., A.T., and J.T. planned and performed the LC‐HRMS/MS method validation. P.S., S.M.S., and J.T. prepared and analyzed the postadministration urine samples. P.S. evaluated the post‐administration urine samples. The original draft of the manuscript was written by P.S., and was carefully reviewed and edited by M.T., A.T., S.M.S., L.K., and S.G. The entire project was supervised by P.S., A.T. and M.T. All authors have read and agreed to the published version of the manuscript.

## Funding

This work was supported by the World Anti‐Doping Agency (25T04MT).

## Conflicts of Interest

The authors declare no conflicts of interest.

## Supporting information


**Table S1:** Mean, minimum, and maximum concentrations of M5‐b metabolite after administration of 1 × 1 μg of LGD‐4033, summarized per time interval with corresponding number of data points.
**Table S2:** Mean, minimum, and maximum concentrations of M5‐b metabolite after administration of 1 × 10 μg of LGD‐4033, summarized per time interval with corresponding number of data points.
**Table S3:** Mean, minimum, and maximum concentrations of M5‐b metabolite after administration of 1 × 50 μg of LGD‐4033, summarized per time interval with corresponding number of data points.
**Table S4:** Mean, minimum, and maximum concentrations of M5‐b metabolite after administration of 5 × 1 μg of LGD‐4033, summarized per time interval with corresponding number of data points.
**Table S5:** Mean, minimum, and maximum concentrations of M5‐b metabolite after administration of 5 × 10 μg of LGD‐4033, summarized per time interval with corresponding number of data points.
**Table S6:** Mean, minimum, and maximum concentrations of M5‐b metabolite after administration of 5 × 50 μg of LGD‐4033, summarized per time interval with corresponding number of data points.
**Figure S1:** Individual urinary excretion profiles (up to 120 h) of M5‐b concentrations following single oral micro‐dose administration of LGD‐4033 (1, 10, and 50 μg), using the original sampling times.
**Figure S2:** Individual urinary excretion profiles (up to 240 h) of M5‐b concentrations following multiple oral micro‐dose administration of LGD‐4033 (1, 10, and 50 μg), using the original sampling times.

## Data Availability

All MS data generated during the current study are available from the corresponding author on reasonable request.
