# Supplementary material for: Quantification of the Carboxylated LGD‐4033 Long‐Term Metabolite in Human Micro‐Dose Excretion Study Urine Samples
Source: Drug Test Anal. 2026 Aug 3;18(10):1321–7. doi: 10.1002/dta.70130 (PMC13628428; doi:10.1002/dta.70130)
Supplement: Supplementary file 1 [file DTA-18-1321-s001.docx]

Supporting Information

**Quantification of the carboxylated LGD-4033 long-term metabolite in human micro-dose excretion study urine samples**

Panagiotis Sakellariou^1*^, Sebastian Marcel Schröder^1^, Jasmin Thelen^1^, Linus Korsmeier^1^, Sven Guddat^1^, Christian Görgens^1^, Andreas Thomas^1^, Mario Thevis^1,2^

^1^ Institute of Biochemistry/Center for Preventive Doping Research, German Sport University Cologne, Cologne, Germany

^2^ European Monitoring Center for Emerging Doping Agents (EuMoCEDA), Cologne/Bonn, Germany

**Overview**

**Table S1**	Mean, minimum, and maximum concentrations of M5-b metabolite after administration of 1 x 1 µg of LGD-4033, summarized per time interval with corresponding number of data points.

**Table S2**	Mean, minimum, and maximum concentrations of M5-b metabolite after administration of 1 x 10 µg of LGD-4033, summarized per time interval with corresponding number of data points.

**Table S3**	Mean, minimum, and maximum concentrations of M5-b metabolite after administration of 1 x 50 µg of LGD-4033, summarized per time interval with corresponding number of data points.

**Table S4**	Mean, minimum, and maximum concentrations of M5-b metabolite after administration of 5 x 1 µg of LGD-4033, summarized per time interval with corresponding number of data points.

**Table S5**	Mean, minimum, and maximum concentrations of M5-b metabolite after administration of 5 x 10 µg of LGD-4033, summarized per time interval with corresponding number of data points.

**Table S6**	Mean, minimum, and maximum concentrations of M5-b metabolite after administration of 5 x 50 µg of LGD-4033, summarized per time interval with corresponding number of data points.

**Figure S1**	Individual urinary excretion profiles (up to 120 h) of M5-b concentrations following single oral micro-dose administration of LGD-4033 (1, 10, and 50 µg), using the original sampling times.

**Figure S2**	Individual urinary excretion profiles (up to 240 h) of M5-b concentrations following multiple oral micro-dose administration of LGD-4033 (1, 10, and 50 µg), using the original sampling times.

**Table S1.** Mean, minimum, and maximum concentrations of M5-b metabolite after administration of 1 x 1 µg of LGD-4033, summarized per time interval with corresponding number of data points.

| **Time (h)** | **Cmean (ng/mL)** | **Cmin (ng/mL)** | **Cmax (ng/mL)** | **Data points** |
| --- | --- | --- | --- | --- |
| 0-2 | 0.33 | 0.04 | 0.56 | 3 |
| 2-6 | 0.19 | 0.06 | 0.34 | 5 |
| 6-12 | 0.13 | 0.04 | 0.26 | 5 |
| 12-24 | 0.07 | 0.02 | 0.25 | 5 |
| 24-36 | 0.04 | 0.01 | 0.08 | 5 |
| 36-48 | 0.04 | 0.02 | 0.10 | 5 |
| 48-72 | 0.04 | 0.03 | 0.05 | 2 |
| 72-96 | 0.02 | - | - | 1 |

**Table S2.** Mean, minimum, and maximum concentrations of M5-b metabolite after administration of 1 x 10 µg of LGD-4033, summarized per time interval with corresponding number of data points.

| **Time (h)** | **Cmean (ng/mL)** | **Cmin (ng/mL)** | **Cmax (ng/mL)** | **Data points** |
| --- | --- | --- | --- | --- |
| 0-2 | 1.90 | 0.63 | 3.79 | 4 |
| 2-6 | 1.63 | 0.19 | 2.66 | 5 |
| 6-12 | 0.90 | 0.17 | 2.10 | 5 |
| 12-24 | 0.52 | 0.05 | 1.39 | 5 |
| 24-36 | 0.22 | 0.02 | 1.41 | 5 |
| 36-48 | 0.14 | 0.02 | 0.45 | 5 |
| 48-72 | 0.05 | 0.01 | 0.10 | 4 |
| 72-96 | 0.06 | 0.01 | 0.14 | 4 |
| 96-120 | 0.03 | 0.02 | 0.04 | 2 |
| 120-144 | 0.02 | 0.01 | 0.02 | 2 |
| 144-168 | 0.04 | 0.04 | 0.04 | 2 |
| 168-192 | 0.01 | - | - | 1 |

**Table S3.** Mean, minimum, and maximum concentrations of M5-b metabolite after administration of 1 x 50 µg of LGD-4033, summarized per time interval with corresponding number of data points.

| **Time (h)** | **Cmean (ng/mL)** | **Cmin (ng/mL)** | **Cmax (ng/mL)** | **Data points** |
| --- | --- | --- | --- | --- |
| 0-2 | 29.1 | 2.91 | 82.6 | 4 |
| 2-6 | 19.5 | 6.87 | 40.6 | 4 |
| 6-12 | 5.62 | 1.01 | 13.6 | 4 |
| 12-24 | 3.59 | 1.15 | 6.28 | 4 |
| 24-36 | 2.93 | 0.27 | 8.37 | 5 |
| 36-48 | 1.35 | 0.23 | 6.27 | 5 |
| 48-72 | 1.37 | 0.02 | 2.64 | 4 |
| 72-96 | 1.20 | 0.09 | 5.05 | 5 |
| 96-120 | 0.51 | 0.01 | 1.89 | 5 |
| 120-144 | 0.24 | 0.03 | 0.75 | 4 |
| 144-168 | 0.10 | 0.02 | 0.32 | 4 |
| 168-192 | 0.27 | - | - | 1 |
| 192-216 | 0.17 | 0.01 | 0.33 | 2 |
| 216-240 | 0.24 | 0.03 | 0.75 | 2 |
| 240-264 | 0.12 | 0.02 | 0.23 | 2 |
| 264-288 | 0.02 | - | - | 1 |
| 288-312 | 0.01 | - | - | 1 |

**Table S4.** Mean, minimum, and maximum concentrations of M5-b metabolite after administration of 5 x 1 µg of LGD-4033, summarized per time interval with corresponding number of data points.

| **Time (h)** | **Cmean (ng/mL)** | **Cmin (ng/mL)** | **Cmax (ng/mL)** | **Data points** |
| --- | --- | --- | --- | --- |
| 0-2 | 0.98 | 0.77 | 1.09 | 3 |
| 2-6 | 0.92 | 0.35 | 2.85 | 5 |
| 6-12 | 0.20 | 0.03 | 0.62 | 5 |
| 12-24 | 0.16 | 0.03 | 0.58 | 5 |
| 24-36 | 0.72 | 0.02 | 3.93 | 5 |
| 36-48 | 0.26 | 0.02 | 0.87 | 5 |
| 48-72 | 0.66 | 0.02 | 3.82 | 5 |
| 72-96 | 0.66 | 0.10 | 1.91 | 5 |
| 96-120 | 0.78 | 0.04 | 3.61 | 5 |
| 120-144 | 0.13 | 0.02 | 0.26 | 5 |
| 144-168 | 0.06 | 0.04 | 0.08 | 4 |
| 168-192 | 0.14 | 0.01 | 0.47 | 4 |
| 192-216 | 0.24 | 0.03 | 0.64 | 3 |
| 216-240 | 0.04 | 0.01 | 0.09 | 3 |
| 240-264 | 0.04 | 0.02 | 0.06 | 2 |

**Table S5.** Mean, minimum, and maximum concentrations of M5-b metabolite after administration of 5 x 10 µg of LGD-4033, summarized per time interval with corresponding number of data points.

| **Time (h)** | **Cmean (ng/mL)** | **Cmin (ng/mL)** | **Cmax (ng/mL)** | **Data points** |
| --- | --- | --- | --- | --- |
| 0-2 | 3.31 | 1.99 | 4.39 | 3 |
| 2-6 | 4.75 | 0.94 | 17.3 | 5 |
| 6-12 | 1.70 | 0.08 | 5.29 | 5 |
| 12-24 | 1.04 | 0.02 | 5.86 | 5 |
| 24-36 | 4.54 | 0.26 | 15.3 | 5 |
| 36-48 | 2.01 | 0.20 | 7.11 | 5 |
| 48-72 | 2.76 | 0.20 | 16.0 | 5 |
| 72-96 | 3.76 | 0.27 | 10.9 | 5 |
| 96-120 | 2.88 | 0.15 | 7.87 | 5 |
| 120-144 | 1.51 | 0.14 | 2.68 | 5 |
| 144-168 | 0.24 | 0.08 | 0.40 | 5 |
| 168-192 | 0.20 | 0.01 | 0.93 | 5 |
| 192-216 | 0.19 | 0.01 | 0.65 | 4 |
| 216-240 | 0.14 | 0.01 | 0.42 | 2 |
| 240-264 | 0.11 | 0.10 | 0.12 | 2 |
| 264-288 | 0.03 | - | - | 1 |
| 288-312 | 0.02 | 0.01 | 0.02 | 2 |
| 312-336 | 0.01 | - | - | 1 |
| 360-384 | 0.02 | - | - | 1 |

**Table S6.** Mean, minimum, and maximum concentrations of M5-b metabolite after administration of 5 x 50 µg of LGD-4033, summarized per time interval with corresponding number of data points.

| **Time (h)** | **Cmean (ng/mL)** | **Cmin (ng/mL)** | **Cmax (ng/mL)** | **Data points** |
| --- | --- | --- | --- | --- |
| 0-2 | 8.93 | 0.03 | 22.3 | 4 |
| 2-6 | 31.9 | 3.33 | 79.3 | 5 |
| 6-12 | 12.8 | 0.33 | 68.9 | 5 |
| 12-24 | 7.61 | 0.78 | 17.8 | 5 |
| 24-36 | 21.0 | 1.15 | 198 | 5 |
| 36-48 | 12.6 | 1.11 | 42.4 | 5 |
| 48-72 | 18.5 | 0.88 | 61.8 | 5 |
| 72-96 | 21.4 | 0.57 | 241 | 5 |
| 96-120 | 25.9 | 0.68 | 182 | 4 |
| 120-144 | 4.33 | 0.76 | 10.4 | 4 |
| 144-168 | 2.34 | 0.48 | 6.42 | 4 |
| 168-192 | 0.69 | 0.29 | 1.08 | 4 |
| 192-216 | 0.72 | 0.12 | 2.04 | 4 |
| 216-240 | 0.50 | 0.04 | 1.23 | 4 |
| 240-264 | 0.29 | 0.01 | 0.94 | 5 |
| 264-288 | 0.10 | 0.03 | 0.19 | 5 |
| 288-312 | 0.12 | 0.03 | 0.21 | 4 |
| 312-336 | 0.13 | 0.02 | 0.37 | 4 |
| 336-360 | 0.11 | 0.09 | 0.13 | 2 |
| 360-384 | 0.06 | 0.05 | 0.06 | 2 |
| 384-408 | 0.04 | - | - | 1 |
| 408-432 | 0.08 | 0.03 | 0.12 | 2 |
| 432-456 | 0.03 | 0.02 | 0.04 | 2 |
| 456-480 | 0.04 | 0.02 | 0.08 | 2 |
| 480-504 | 0.07 | 0.02 | 0.17 | 2 |
| 504-528 | 0.07 | 0.01 | 0.12 | 2 |
| 528-552 | 0.06 | 0.02 | 0.10 | 2 |
| 552-576 | 0.07 | - | - | 1 |
| 576-600 | 0.07 | 0.02 | 0.13 | 2 |
| 600-624 | 0.03 | 0.01 | 0.04 | 2 |
| 624-648 | 0.03 | - | - | 1 |
| 648-672 | 0.08 | - | - | 1 |
| 672-696 | 0.04 | - | - | 1 |
| 696-720 | 0.01 | - | - | 1 |
| 720-744 | 0.05 | - | - | 1 |
| 768-792 | 0.02 | - | - | 1 |
| 792-816 | 0.04 | - | - | 1 |
| 816-840 | 0.03 | - | - | 1 |

**Figure S1.** Individual urinary excretion profiles (up to 120 h) of M5-b concentrations following single oral micro-dose administration of LGD-4033 (1, 10, and 50 µg), using the original sampling times.

**Figure S2.** Individual urinary excretion profiles (up to 240 h) of M5-b concentrations following multiple oral micro-dose administration of LGD-4033 (1, 10, and 50 µg), using the original sampling times.
